# Increased Gal-3BP plasma levels in hospitalized patients infected with SARS-CoV-2

**DOI:** 10.1007/s10238-021-00788-8

**Published:** 2022-01-25

**Authors:** Valentina Gallo, ISERC-Team, Ana Reis, André Miranda, Carolina Martins, Cláudia Serre-Miranda, Claudia Nobrega, Carolina S. Silva, Helena Sarmento, Jorge Cotter, João Canto-Gomes, Joana Palha, Pedro Peixoto, Palmira Barreira-Silva, João Carlos Sousa, Margarida Correia-Neves, Mariana Formigo, Neide Vieira, Pedro G. Cunha, Susana Roque, Roberta Gentile, Giovanni Antonini, Stefano Iacobelli

**Affiliations:** 1 Department of Sciences, Roma Tre University, Rome, Italy; 2 Biostructures and Biosystems National Institute (INBB), Rome, Italy; 3 MediaPharma Srl, Chieti, Italy

**Keywords:** Gal-3BP, Biomarkers, SARS-CoV-2, COVID-19

## Abstract

Coronavirus disease 2019 (COVID-19) has quickly turned into a health, financial and societal problem globally. The complex pathogenesis of severe acute respiratory syndrome coronavirus centers on the unpredictable clinical progression of the disease, which may evolve abruptly and results in critical and life-threatening clinical complications. Effective laboratory biomarkers that can classify patients according to risk of progression to severe disease are essential for ensuring timely treatment. Gal-3BP is a human secreted protein with innate immune functions, which is upregulated in viral infections, promotes inflammation and has been shown to induce IL-6 expression. In this study, Gal-3BP plasma levels were measured retrospectively in a cohort of 84 hospitalized COVID-19 patients. These were classified as having either “non-severe” or “severe” disease. Compared to healthy controls, Gal-3BP plasma levels were markedly increased in COVID-19 patients (*P* < 0.0001). Moreover, the levels were higher in severe than in non-severe patients (*P* < 0.05). As expected, patients with severe disease had plasma levels of IL-6 higher than patients with non-severe disease (*P* < 0.01). In non-severe disease patients, Gal-3BP levels collected at a late stage (13.3 + 5.7 days after the first positive PCR result) were significantly lower than those collected at an early stage (4.2 + 2.9 days form the first positive PCR result). Larger prospective analyses are needed to strength our understanding of the prognostic utility of Gal-3BP in COVID-19 patients.

## Introduction

Severe acute respiratory syndrome coronavirus 2 (SARS-CoV-2) is the strain of coronavirus that causes the novel coronavirus disease 2019 (COVID-19), which is the respiratory illness responsible for the global COVID-19 pandemic. By November 2021, nearly 257 million infections and over 5 million deaths have been reported globally so far [1].

The spectrum of COVID-19 disease can range from asymptomatic infection to severe pneumonia with acute respiratory distress syndrome. Effective laboratory biomarkers that can early classify infected patients according to the risk of progression to severe disease could facilitate clinical management and early therapeutic intervention.

Although the pathogenesis of COVID-19 severity remains unclear, numerous studies have shown an increase in the level of IL-6 and other proinflammatory cytokines in COVID-19 hospitalized patients and its association with the severity of the disease and mortality [2–4].

Gal-3BP (Uniprot ID–Q08380), also known as 90 K, Mac-2 BP or LGALS3BP is a secreted protein belonging to the macrophage scavenger receptor cysteine-rich domain superfamily, which was originally identified by two independent research groups while aiming to study proteins secreted in vitro by cancer cell lines [5, 6]. Functionally, Gal-3 BP activates antiviral innate immune responses, promotes inflammation [7] and induces IL-6 expression and secretion [8]. Previously, Gal-3BP has been found to be elevated in the serum of patients infected with various kinds of viral infections, including human immunodeficiency virus (HIV) [9] and hepatitis C virus [10]. Importantly, in HIV-infected patients that were initially symptom-free, Gal-3BP levels were higher in those who progressed to Acquired Immunodeficiency Syndrome [11]. In the present study, we examined the relationships between Gal-3BP plasma levels and severity of the disease in a cohort of hospitalized COVID-19 patients.

## Methods

The present study retrospectively enrolled 84 confirmed COVID-19 patients who were hospitalized at Hospital Senhora da Oliveira Guimarães (Portugal) from April 2020 to January 2021. The diagnosis was confirmed by detecting SARS-CoV-2 RNA by RT-qPCR in oro-nasopharyngeal swab samples. Also, 24 age- and gender-matched healthy individuals (blood donors) were recruited as controls. The mean period of hospitalization was 18.4 ± 10.1 days (median 17 days; range 4–50 days). Patients were classified as having either “non-severe” or “severe” disease according to the worst clinical presentation achieved during hospitalization. The “non-severe” group included patients characterized by the MaxFiO2 (fraction of inspired oxygen) of either ≤ 28% or > 28%, excluding severe cases. The “severe” group included patients who required advanced respiratory support, including mechanical ventilation (invasive or noninvasive) or high-flow nasal cannula within 12 h of admission, and/or were admitted to Intensive Care Unit (ICU) and/or died during hospitalization. Data were anonymized for analysis and the study was approved by the Ethics Committee of the Hospital Senhora da Oliveira. The informed consent was prepared according to the Declaration of Helsinki principles, the Oviedo Convention and the General Data Protection Regulation–Regulation (EU) 2016/679.

Plasma samples were collected in two time periods after hospitalization: “early” samples, collected 4.7 ± 3.8 days after the first positive PCR result and “late” samples, collected 13.4 ± 6.5 days after the first positive PCR result.

Gal-3BP plasma concentrations were measured by a sandwich ELISA according to the previously described procedure [12]. The murine anti-human Gal-3BP SP-2 antibody and its humanized variant 1959 (both from MediaPharma Srl, Italy) were used as capture and detection antibody, respectively; Gal-3BP (MediaPharma Srl, Italy) was used as standard antigen for the construction of the calibration curve. IL-6 plasma concentrations were measured by the commercial Salivary IL-6 ELISA kit (Salimetrics, 1-3602-5, State College, PA, USA).

Statistical analysis was performed using Microsoft Excel and the Real Statistics Resource Pack software (Release 7.6). Copyright (2013–2021) Charles Zaiontz. www.real-statistics.com. Student’s *t* test and two-way ANOVA were used for calculating the *P* values; sensitivity, specificity, receiver operating characteristic curve (ROC) and area under curve (AUC) were determined for plasma Gal-3BP.

## Results

The baseline clinical characteristics of COVID‐19 patients are shown in Table 1. Except for age, which was significantly older in patients classified as severe, all the parameters analyzed did not differed significantly between the two groups. In severe patients, mechanical ventilation was adopted in 38%; 31% went to ICU and 56% died.

**Table 1:** Clinical characteristics of patients according to the severity of COVID-19 disease

Parameter	Non-severe	Severe
Total number	45	39
Men	47	62
Average age*	68.7 ± 16	76.8 ± 12
Lack of autonomy	11%	21%
Hypertension	69%	72%
Diabetes	31%	46%
Immunosuppression	13%	13%
Neoplasia	9%	10%
Autoimmune disorder	2%	0%
HIV	0%	0%
Transplant	0%	3%
Other pulmonary disease	11%	23%
Smoking habit	4%	13%
Obesity	27%	13%

The severe group included patients who required advanced respiratory support, including mechanical ventilation (invasive or noninvasive) or high-flow nasal cannula within 12 h of admission, and/or were admitted to ICU and/or died during hospitalization

Patients were classified as severe 5.0 ± 4.6 days after hospitalization (median 3.0 days, range 1–15 days)

*P < 0.05

**Fig. 1:**
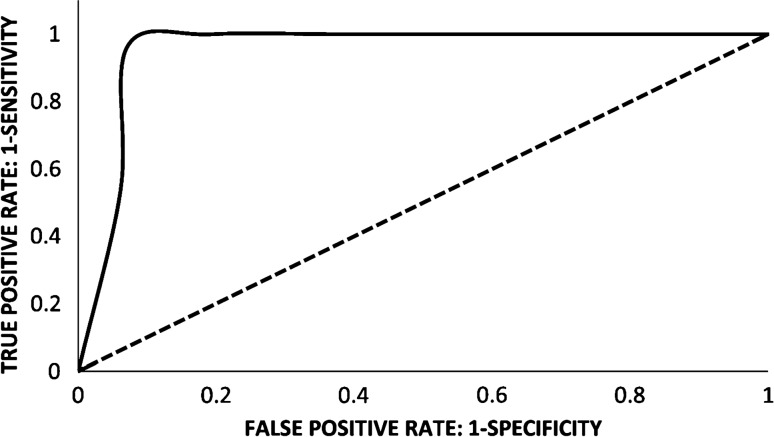
ROC curve of plasma Gal-3BP levels in hospitalized COVID‐19 patients compared with healthy controls (AUC = 0.97), using a cut-off value of 2.75 µg/ml

**Fig. 2:**
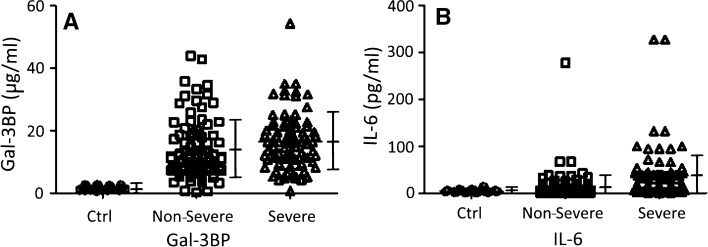
Plasma levels of Gal-3BP (**a**) and IL-6 (**b**) in healthy controls (Ctrl) and in hospitalized COVID-19 patients classified as “Non Severe” or “Severe”. ANOVA test showed statistically significant differences of Gal-3BP levels between “Non Severe” and “Severe” patients (*P* < 0.05) and IL-6 levels between “Non Severe” and “Severe “patients (*P* < 0.01). Vertical bars indicate Means ± Standard Deviations

Compared to healthy controls, Gal-3BP levels in plasma samples of hospitalized COVID-19 patients were markedly increased (14.8 ± 9.5 µg/ml vs. 1.45 ± 0.6 µg/ml; *P* < 0.0001). Using a statistically optimal cut-off value of 2.75 µg/ml, a Receiver Operating Characteristic (ROC) curve of Gal-3BP levels in healthy controls versus COVID-19 patients was generated with an Area Under the Curve (AUC) of 0.97 (with 95% confidence interval from 0.99 to 0.95) (Fig. 1). Furthermore, Gal-3BP levels were higher in severe than non-severe patients (16.4 ± 9.8 µg/ml, vs. 13.3 ± 8.9 µg/ml; *P* < 0.05; Fig. 2a). Similarly, plasma levels of IL-6 were much higher in severe patients (37.7 ± 52.4 pg/ml) than in non-severe patients (14.5 ± 31.3 pg/ml; *P* < 0.01; Fig. 2b).

**Fig. 3:**
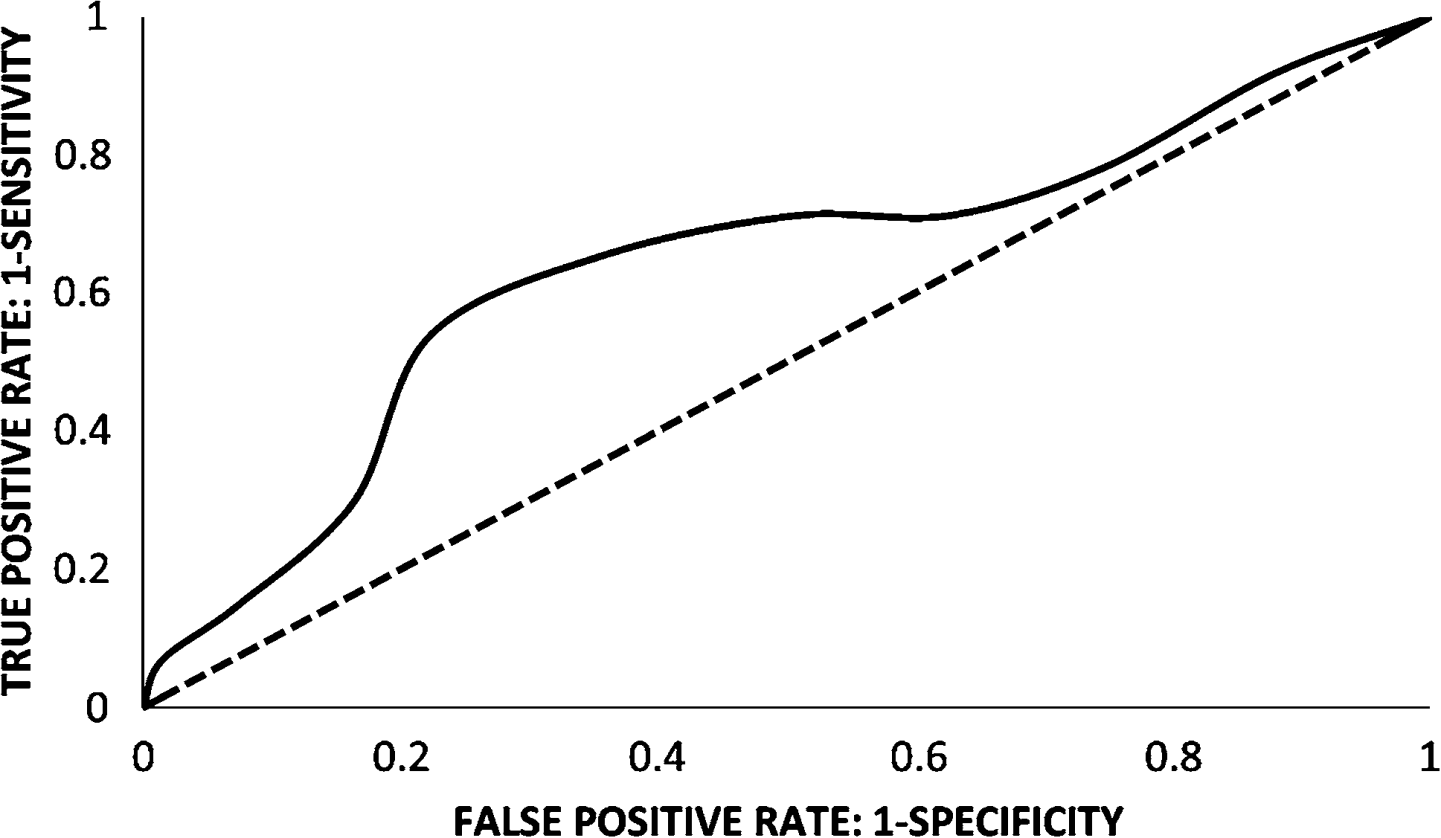
ROC curve of plasma Gal-3BP levels in hospitalized COVID‐19 patients classified as “Severe” compared with patients classified as “Non Severe” (AUC = 0.68), using a cut-off value of 10.5 µg/ml

Using a statistically optimal cut-off value of 10.5 µg/ml, a ROC curve of Gal-3BP levels in non-severe versus severe patients was drawn with an AUC of 0.68 (with 95% confidence interval from 0.65 to 0.71) (Fig. 3). This cut off correctly classified 34 out of 39 patients as severe (Positive Predictive Value, PPV = 57% and Specificity = 42%) and 19 out of 45 patients as non-severe (Negative Predictive Value, NPV = 79% and Sensitivity = 87%), with an Accuracy of 63%. Although the difference between Gal-3BP levels in non-severe versus severe patients was relatively low, it is worth noting that NPV and Sensitivity were relatively high.

Finally, Gal-3BP levels varied during hospitalization. As shown in Table 2, a significant decrease of Gal-3BP levels was observed in samples collected at late stage compared to those collected at early stage from patients classified as non-severe, whereas in patients classified as severe the protein remained stable, irrespective of the time of collection (Table 2).

**Table 2:** Time-dependent Gal-3BP plasma levels in COVID-19 hospitalized patients

	Early sampling		Late sampling		Hospitalization time (days)
	Days	Gal-3BP (µg/ml)	Days	Gal-3BP (µg/ml)	
All patients	4.7 ± 3.8	16.4 ± 9.6	13.4 ± 6.5	12.4 ± 9.7*	19.5 ± 10.6
Non-severe	4.2 ± 2.9	15.4 + 10.5	13.3 + 5.7	8.9 + 5.9**	19.7 ± 11.5
Severe	5.1 ± 3.2	18.0 ± 8.0	13.6 ± 8.4	15.6 ± 11.0***	19.0 ± 9.2

Difference in late Gal-3BP between “non-severe” and “severe” patients *P* = 0.0005

*Difference between early Gal-3BP and late Gal-3BP in all patients *P* = 0.016

**Difference between early Gal-3BP and late Gal-3BP in “non-severe” patients *P* = 0.0013

***Difference between early Gal-3BP and late Gal-3BP in “severe” patients, not significant

## Conclusions

The present study shows that Gal-3BP is associated with COVID-19. We found highly increased plasma levels of Gal-3BP in hospitalized COVID-19 patients compared to healthy controls. Furthermore, the levels of the protein were higher in patients with severe than in those with non- severe disease.

The relationships between Gal-3BP and disease severity in COVID-19 patients have been investigated in recent studies. Using a ultra-high-throughput serum and plasma proteomics, an about 3.4-fold increase of Gal-3BP levels was detected in COVID-19 patients during early hospitalization and claimed as the one of potential biomarkers that was upregulated with increasing disease severity [13]. In another study, Gal-3BP was found markedly elevated in blood samples of patients admitted to ICU for COVID-19 versus those non-admitted to ICU or those admitted to ICU for non-COVID-19 related sepsis [14]. Importantly, in this latter study Gal-3BP was identified as an interaction partner of SARS-CoV-2 spike glycoprotein whose overexpression inhibited spike-pseudoparticle uptake and spike-induced cell–cell fusion in vitro [14]. Finally, a longitudinal analysis of COVID-19 patients during hospitalization revealed that Gal-3BP and other proteins of the innate immune system mediators such as complement factors C2, C9, C4BPA, alpha-1-acid glycoprotein 1 (ORM1) and monocyte differentiation antigen CD14, were upregulated early during hospitalization but decreased at later times [15].

Our data on relationships between Gal-3BP and disease severity were only marginally significant. This could be due to the long temporal interval between the first positive PCR result and the Gal-3BP plasma sampling, which in about half of the cases was taken at a late stage (i.e., 13.4 ± 6.5 days) after the first positive PCR result, which, in turn, was obtained about 2 days after symptoms onset (on average 2.5 ± 2.7 days). Many events should have occurred after initial elevation of Gal-3BP which might have triggered mechanisms linked to progression to a severe disease. Indeed, Gal-3BP levels were found to be high at early stage and decreased over time in patients classified as non-severe, whereas they remained stably high in those classified as severe (Table 2). This observation and the results of previous studies [13, 15] seem to indicate that a prognostic information of Gal-3BP is obtained when the protein is measured twice during the course of the infection: early, soon after the first positive PCR result and at a later stage, i.e., 2 weeks after the first positive PCR result. A significant reduction of Gal-3 BP levels in the second versus the first measurement should be indicative of a non-progressive disease.

Currently, no effective treatment is available for COVID-19 patients. There is a general consensus that treating patients early in the course of the infection would speed their recovery and reduce the likelihood that they develop severe outcomes [16]. Therefore, early identification of patients at risk of developing severe disease is of outmost importance. High levels of diverse inflammatory cytokines have been associated with the risk of developing severe of COVID-19 disease [17]. Of these, IL-6 has been proposed as the most accurate predictor of disease course and mortality [2, 18]. In this context, data have been presented to show that Gal-3BP is able to stimulate expression and secretion of IL-6 in multiple different cell types [8]. Hence, a model can be proposed thereby SARS-CoV-2 induces Gal-3BP in infected cells, which, in turn stimulates production and secretion of IL-6, a major trigger of inflammation and cytokine storm.

Limitations of this study include its single-centered retrospective nature, the small sample size, and late blood sampling for Gal-3BP assessment. Future efforts focused on large prospective analyses will strengthen our understanding of the prognostic utility of Gal-3BP.

## Data Availability

The dataset supporting the conclusions of this article is included within the article as additional file.

## References

[1] https://www.worldometers.info/coronavirus

[2] Zhang J, Hao Y, Ou W (2020). Serum interleukin-6 is an indicator for severity in 901 patients with SARS-CoV-2 infection: a cohort study. J Transl Med.

[3] Giannakodimos I, Gkountana GV, Lykouras D, Karkoulias K, Tsakas S (2021). The role of interleukin-6 in the pathogenesis, prognosis and treatment of severe COVID-19. Curr Med Chem.

[4] Liu X, Wang H, Shi S (2021). Association between IL-6 and severe disease and mortality in COVID-19 disease: a systematic review and meta-analysis. Postgrad Med J.

[5] Iacobelli S, Arnò E, D’Orazio A (1986). Detection of antigens recognized by a novel monoclonal antibody in tissue and serum from patients with breast cancer. Cancer Res.

[6] Linsley PS, Horn D, Marquardt H (1986). Identification of a novel serum protein secreted by lung carcinoma cells. Biochemistry.

[7] Xu G, Xia Z, Deng F (2019). Inducible LGALS3BP/90K activates antiviral innate immune responses by targeting TRAF6 and TRAF3 complex. PLoS Pathog.

[8] Silverman AM, Nakata R, Shimada H, Sposto R, DeClerck YA (2012). A galectin-3-dependent pathway upregulates interleukin-6 in the microenvironment of human neuroblastoma. Cancer Res.

[9] Natoli C, Iacobelli S, Ghinelli F (1991). Unusually high level of a tumor-associated antigen in the serum of human immunodeficiency virus-seropositive individuals. J Infect Dis.

[10] Artini M, Natoli C, Tinari N (1996). Elevated serum levels of 90K/MAC-2 BP predict unresponsiveness to alpha-interferon therapy in chronic HCV hepatitis patients. J Hepatol.

[11] Iacobelli S, Ullrich A, Tinari N (1995). The 90K tumor-associated antigen and clinical progression in human immunodeficiency virus infection. J Acquir Immune Defic Syndr Hum Retrovirol.

[12] Gallo V, Lai A, Pasquo A (2020). Surface-enhanced Raman scattering (SERS)-based immunosystem for ultrasensitive detection of the 90K biomarker. Anal Bioanal Chem.

[13] Messner CB, Demichev V, Wendisch D (2020). Ultra-high-throughput clinical proteomics reveals classifiers of COVID-19 infection. Cell Syst.

[14] Gutmann C, Takov K, Burnap SA (2021). SARS-CoV-2 RNAemia and proteomic trajectories inform prognostication in COVID-19 patients admitted to intensive care. Nat Commun.

[15] Geyer PE, Arend FM, Doll S (2021). High-resolution serum proteome trajectories in COVID-19 reveal patient-specific seroconversion. EMBO Mol Med.

[16] Kim PS, Read SW, Fauci AS (2020). Therapy for early COVID-19a critical need. JAMA.

[17] Zeng F, Huang Y, Guo Y (2020). Association of inflammatory markers with the severity of COVID-19: a meta-analysis. Int J Infect Dis.

[18] Zhou F, Yu T, Du R (2020). Clinical course and risk factors for mortality of adult inpatients with COVID-19 in Wuhan, China: a retrospective cohort study. Lancet.

